# From BIDS-Formatted EEG Data to Sensor-Space Group Results: A Fully Reproducible Workflow With EEGLAB and LIMO EEG

**DOI:** 10.3389/fnins.2020.610388

**Published:** 2021-01-11

**Authors:** Cyril R. Pernet, Ramon Martinez-Cancino, Dung Truong, Scott Makeig, Arnaud Delorme

**Affiliations:** ^1^Centre for Clinical Brain Sciences, The University of Edinburgh, Edinburgh, United Kingdom; ^2^Swartz Center for Computational Neurosciences, University of California, San Diego, San Diego, CA, United States; ^3^Centre de Recherche Cerveau et Cognition, Université Toulouse III – Paul Sabatier, Toulouse, France; ^4^Centre National de la Recherche Scientifique, Centre de Recherche Cerveau et Cognition, Toulouse, France

**Keywords:** brain imaging data structure, preprocessing algorithm, linear models, reproducibility and tools, EEGLAB toolbox, LIMO EEG

## Abstract

Reproducibility is a cornerstone of scientific communication without which one cannot build upon each other’s work. Because modern human brain imaging relies on many integrated steps with a variety of possible algorithms, it has, however, become impossible to report every detail of a data processing workflow. In response to this analytical complexity, community recommendations are to share data analysis pipelines (scripts that implement workflows). Here we show that this can easily be done using EEGLAB and tools built around it. BIDS tools allow importing all the necessary information and create a study from electroencephalography (EEG)-Brain Imaging Data Structure compliant data. From there preprocessing can be carried out in only a few steps using EEGLAB and statistical analyses performed using the LIMO EEG plug-in. Using Wakeman and Henson (2015) face dataset, we illustrate how to prepare data and build different statistical models, a standard factorial design (faces ^∗^ repetition), and a more modern trial-based regression approach for the stimulus repetition effect, all in a few reproducible command lines.

## Introduction

As data analyses become more and more complex, it has been advocated that clear workflows and all of the parameters used in their implementation should be reported in order to increase reproducibility (Pernet et al., 2020). It is, however, difficult to concisely report a workflow and maybe even impossible given hidden parameters built into the algorithms we use. One solution is to report in detail the workflow and share the corresponding pipelines—thus only having to communicate key algorithm details. Such pipelines and/or tools to build pipelines have been developed in recent years (see, e.g., Bigdely-Shamlo et al., 2015; Andersen, 2018; Jas et al., 2018; Niso et al., 2019; Meunier et al., 2020) and here we describe tools developed around EEGLAB (Delorme and Makeig, 2004) which also allow creating a fully reproducible pipeline from raw data to group results, with an example to sensor space analysis.

**FIGURE 1:**
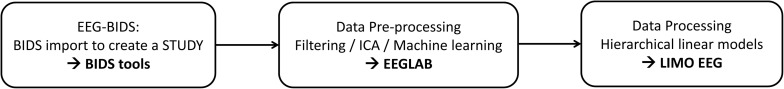
Workflow overview for data processing with EEGLAB.

New data formatting conventions and public repositories for electroencephalography (EEG) data have recently been developed and made available to the science community. In particular, the Brain Imaging Data Structure (Gorgolewski et al., 2016) and its EEG extension (Pernet et al., 2019) allow defining important EEG metadata information, such as additional event information, electrode positions, and experimental conditions. This makes data aggregation from different experiments and analysis automation using standardized pipelines easier. Here we present a fully reproducible workflow (Figure 1) from raw data to group results using open data and we document and share the pipeline.

EEGLAB (Delorme and Makeig, 2004) is the most commonly used platform for EEG data analysis (Hanke and Halchenko, 2011; Martínez-Cancino et al., 2020) and all steps proposed can also be reproduced from the user interface. We refer to the extensive EEGLAB online user manual for GUI operations, and simply point that functions called by interface operations in EEGLAB are saved into the EEG.history field, thus allowing to copy/paste the underlying code to build a different pipeline than the one proposed here. EEGLAB is used here in conjunction with newly developed EEG-BIDS^1^ tools which allow automatically importing and create a STUDY (see Figure 1) for data that follow the EEG-Brain Imaging Data Structure and with the LIMO-EEG toolbox which allows statistical analyses using robust hierarchical linear models (Pernet et al., 2011). The overarching goal of this analysis is to show that it is possible to use a simple pipeline to process “raw” data and perform complex statistical analyses using EEGLAB and LIMO.

## Methods

### Open Data

The pipeline was executed using the EEG data from the multimodal face dataset (Wakeman and Henson, 2015). EEG (70 channel Easycap^2^) and ECG data were extracted from the binary MEG.fif files that combined MEG, EEG, ECG channels, event markers were time corrected (−34 ms) and electrode positions re-oriented to fit the head coordinate system. Out of the 19 participants, participant 1 was removed because of channels digitization errors leading to 18 participants. Data were then organized using EEG-BIDS and archived for download at https://openneuro.org/datasets/ds002718. This dataset is thus a modified and simplified version of the original dataset, with a subset of this original dataset itself made available by authors at https://openneuro.org/datasets/ds000117. In the original data, there were multiple runs for each subject, so we have merged them to make it simpler for users. We have also formatted scanned electrode positions so they are available within the BIDS dataset. We have corrected event latencies, renamed some events, and added information on events and stimulus repetition. We also resampled the data to 250 Hz so it is not as large and can be used for tutorials. All modifications to the original data documented in the readme file of BIDS ds002718 and conversion script are also made available.

The experiment consisted of the presentation of 300 grayscale photographs of familiar and unfamiliar faces, along with their scrambled versions, all repeated twice. Trials started by a fixation cross lasting 400–600 ms, followed by stimuli lasting from 800 to 1000 ms and the repetition of the images occurred either immediately or after 5 or 15 stimuli, leading to a range of time intervals between repeats. Participants had to perform an orthogonal symmetry judgment task ensuring attention to each stimulus.

### Software

Analyses are performed using Matlab 2020a (The Mathworks, Inc.^3^) on Windows or Mac OSx with the Statistical and Machine Learning Toolbox installed, along with EEGLAB^4^ (v2020.0) and its BIDS tool^5^ (v3.5) and LIMO EEG^6^ (v3) plugins—both of them available through the EEGLAB plugin manager.

### BIDS-Import

**FIGURE 2:**
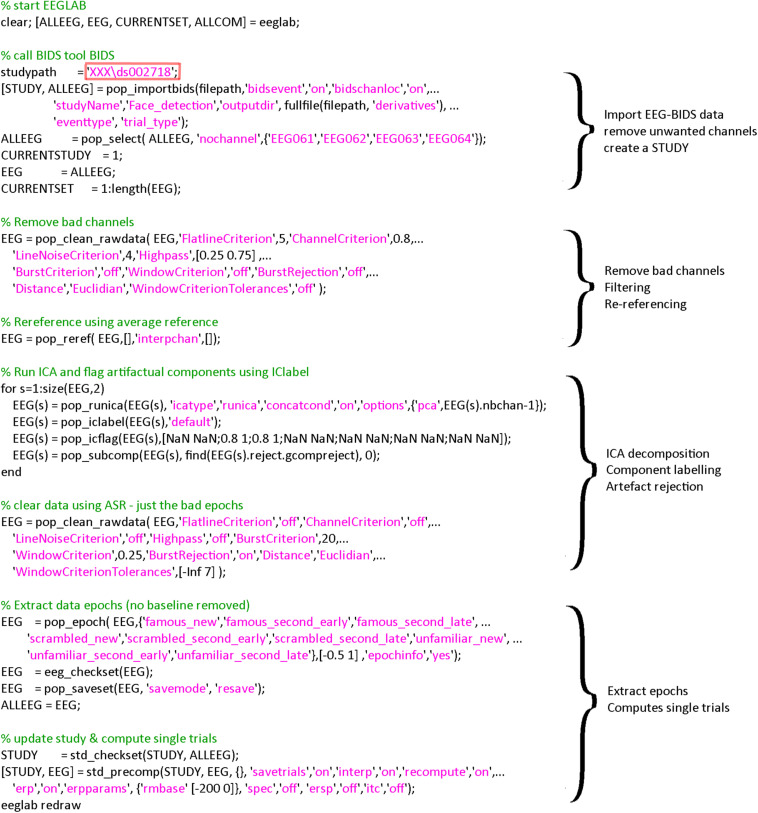
Code used for importing and preprocessing. The region highlighted in red must be changed by the user to point to the location of the data on his/her hard drive. A version of this code that can be copied and pasted is available in the LIMO MEEG repository: https://github.com/LIMO-EEG-Toolbox/limo_ meeg/tree/master/resources/from_bids2stats.m. Note that this part of code was automatically generated from EEGLAB and EEGLAB plugins menus by using the command history function eegh- and then edited for clarity.

The EEGLAB BIDS plugin allows importing BIDS datasets as EEGLAB studies. The plugin allows overwriting events and channel information contained in the raw EEG data—we are using both of these options here. This allows, for example, to define more precise events and channel information—such as channel locations derived from scanned Polhemus positions. The plugin also allows selecting specific fields in the BIDS event file (in this case we used “trial_type” to be mapped to the EEGLAB “type” field). This is reflected in the call to *pop_importbids.m* function in Figure 2.

### Fully Automated Preprocessing

We present here a workflow with minimal preprocessing (Figure 2), which is a set of steps that removes common artifacts without trying to optimize any particular data features, using fully automated methods. Note that, as mentioned earlier, the goal is to present a fully reproducible workflow of ERP analyses with EEGLAB, and not to reproduce or replicate Wakeman and Henson (2015). As such, our preprocessing differs from Wakeman and Hensons’ workflow which, in any case, was primarily designed for the associated MEG data, starting with Signal Space Separation for noise removal (Taulu and Simola, 2006), then bad channel removal, and notch filtering.

**FIGURE 3:**
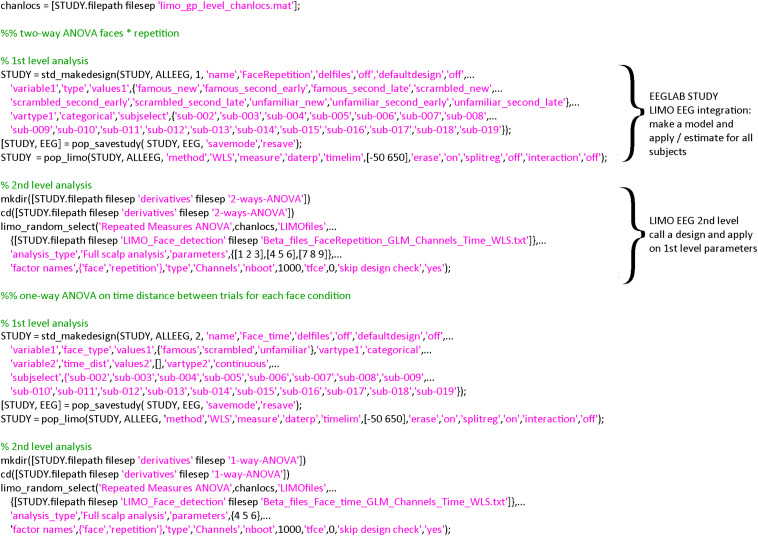
Code snippet for the two statistical models proposed. In both cases, only three steps are necessary: (1) create a statistical design (std_makedesign.m), (2) compute model parameters for each subject (pop_limo.m), and (3) perform the group level analysis (limo_random_select.m). Additional contrasts and figures/plots shown in the results section are also available in command lines, see https://github.com/LIMO-EEG-Toolbox/limo_meeg/tree/master/resources/from_bids2stats.m for details.

As explained above, the code underlying the different steps can be obtained from EEG.history when using the GUI to process subjects. First, bad channels are removed and data filtered at 0.5 Hz using *clean_rawdata* plugin of EEGLAB (v2.2) and the *pop_clean_rawdata.m* function (transition band [0.25 0.75], bad channels defined as a channel exhibiting a flat line of at least 5 s and/or correlation to their robust estimate based on other channels below 0.8). Second, data are re-referenced to the average (*pop_reref.m*) and submitted to an independent component analysis (*pop_runica.m* using the runica algorithm and a reduction in rank to the number of channels −1 to account for average reference). Third, each component is automatically labeled using ICLabel (Pion-Tonachini et al., 2019), rejecting components labeled as eye movements and muscle activity above 80% probability. Finally, continuous data are further cleaned if their power deviated too much from the rest of the data using Artifact Subspace Reconstruction (ASR) algorithm and a 20 burst detection criteria threshold (Kothe and Jung, 2016; Chang et al., 2018) thus taking care of remaining (e.g., line noise, trends) artifacts (*pop_clean_rawdata.m*, burst criterion 20). Note that the data are not corrected by ASR as we only use here the portion of the algorithm detecting bad portions of data and remove them. Also, there is no low-pass filtering since power-line noise is removed via ASR, leaving possible higher frequencies in the otherwise low frequency of visual the ERPs. All parameters are indicated in Figure 3.

### Statistical Modeling

From the clean continuous data, epochs are created by extracting data snippets time-locked (from −500 ms to 1 s) to the face presentation events (*pop_epoch.m*) and designs created within the EEGLAB STUDY. A STUDY in EEGLAB is a structure that contains all the information about the data and metadata allowing to create any experimental designs. Here, we made two designs (*std_makedesigns.m*): one that recreates the faces vs. scrambled effect described by Wakeman and Henson (2015) and one that tests if the time between repetitions of the same stimuli influences the event-related potentials (ERPs—see section “Results” for more details on the statistical models). From first level estimates, repeated measures ANOVA were computed at the group level (*limo_random_select.m*). The goal is to illustrate the flexibility of linear models as implemented in LIMO EEG and ease to create such models using EEGLAB STUDY (Figure 3).

## Results

### Model 1: Coding Conditions Across Trials

At the first level, data from each participant were modeled with nine experimental conditions: famous faces, famous faces repeated immediately, famous faces repeated late, scrambled faces, scrambled faces repeated immediately, scrambled faces repeated late, unfamiliar faces, unfamiliar faces repeated immediately, unfamiliar faces repeated late, and a weighted least squares solution was used to obtain parameter estimates of each condition. At the second level, a repeated measure ANOVA (generalized Hotelling T^2^) was conducted on beta estimates with “faces” and “repetition” as factors. Statistical significance was assessed using spatial–temporal clustering (Maris and Oostenveld, 2007; C.R. Pernet et al., 2015).

**FIGURE 4:**
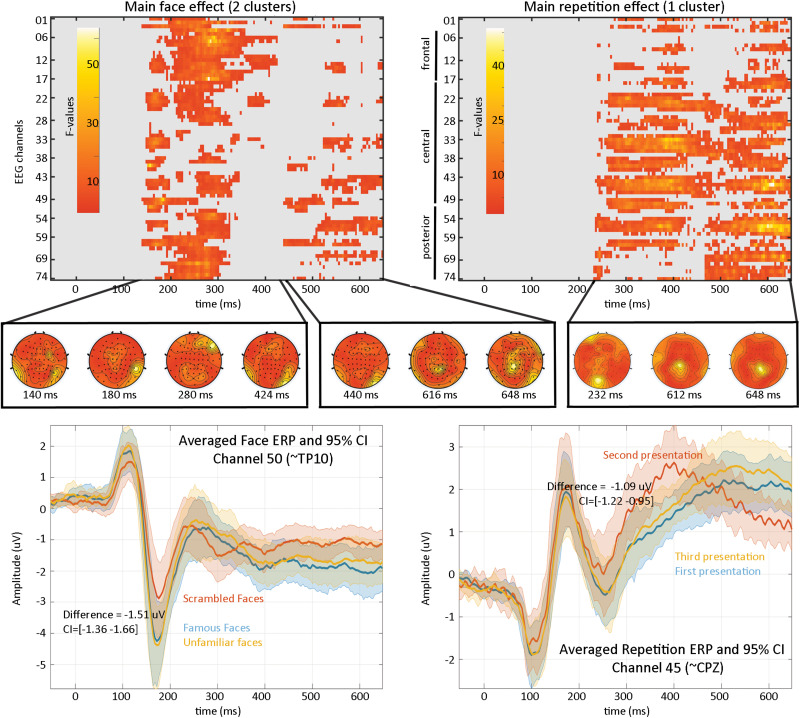
Results from the 2*2 ANOVA analysis. At the top are the main effects for faces and repetition, computed over the whole sensor space. In the middle is the topographical representation of F-values for each significant cluster. At the bottom are displayed the ERP (the mean of the participants weighted means) at channel 50 for faces illustrating the negative face component peaking here at 180 ms and at channel 45 for the repetition effect.

Results (Figure 4) revealed a significant effect of face type with two clusters (cluster 1 starts at 140 ms and ends at 424 ms, maximum F values 64.1281 at 280 ms on channel EEG017, corrected *p*-value 0.002; cluster 2 starts at 440 ms and ends at 648 ms, maximum F value 17.6071 at 616 ms on channel EEG057, corrected *p*-value 0.032) and a significant repetition effect with one cluster (cluster starts at 232 ms and ends at 648 ms, maximum F value 51.3596 at 612 ms channel EEG045, corrected *p*-value 0.001). No significant interaction was observed. As such, the main effect of faces replicates Wakeman and Henson (2015) results who observed “a negative deflection peaking around 170 ms (“N170” component) larger for faces than scrambled faces, which does not differ for familiar and unfamiliar faces. Around 250 ms, a slower potential shift distinguishes familiar and unfamiliar faces until the end of the epoch.” Here we observed higher response at 140 ms (P1 component) for familiar and unfamiliar faces than scrambled faces not previously reported, followed by the same N170 effect—although weaker than reported (from their Figure 1, the face effect is around ∼4 μV while we observed a difference of ∼1.5 μV), likely due to differences in preprocessing (see section “Fully Automated Preprocessing”) and processing (i.e., LIMO EEG used a hierarchical linear model with weighted least squares parameter estimate per subjects while Wakeman and Henson averaged trials per subjects). From 250 ms, we also observed a slow potential separating familiar from unfamiliar faces.

### Model 2: Regressing the Time Between Stimulus Presentation From Trial to Trial

**FIGURE 5:**
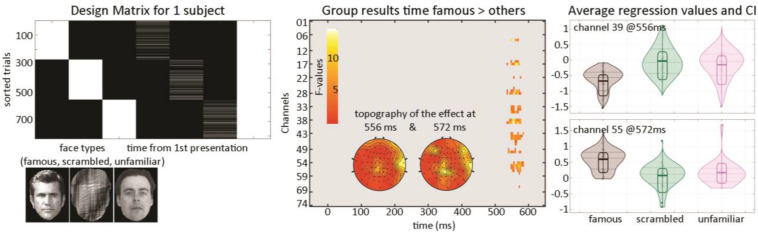
Illustration of 1st level modeling and results from the group level analysis. The design matrix (left) is set up automatically for each participant from EEGLAB study coding here the face types and for each face type, the time delay between repeats of the same stimulus. Results (middle and right) show a late modulation mostly over central electrodes.

At the first level, data from each participant were modeled with three experimental conditions (famous faces, scrambled faces, and unfamiliar faces) along with the time between the repetition of each stimulus (Figure 5) and a weighted least squares solution used to obtain parameter estimates of each condition. At the second level, a repeated measure ANOVA (generalized Hotelling T^2^) was conducted on beta estimates of the time regressors with “faces” as factors, and a *post hoc* contrast computed, testing if the effect of time for famous faces differed from other stimuli. Statistical significance was again assessed using spatial–temporal clustering.

The *post hoc* contrast revealed a significantly stronger modulation of ERP as time passed between famous faces than for other stimuli (Figure 5—cluster 1 starts at 536 ms and ends at 576 ms, maximum 15.1112 at 556 ms channel EEG039, corrected *p*-value 0.031; cluster 2 starts at 548 ms and ends at 584 ms, maximum 9.58689 at 572 ms channel EEG055, corrected *p*-value 0.033). This suggests that there is an interaction face type by repetition such as late ERPs vary as a function of time between the first/second and third repetition for famous faces but not unfamiliar or scrambled faces.

## Discussion

Following community guidelines (Pernet et al., 2020), we have implemented necessary changes and new tools allowing to create fully reproducible workflows with EEGLAB. The pipeline for the presented analysis is available at https://github.com/LIMO-EEG-Toolbox/limo_meeg/tree/master/resources/from_bids2stats.m and further designs presented on the LIMO MEEG GitHub website (via the user interface and command-line alike https://github.com/LIMO-EEG-Toolbox/limo_tools/wiki).

A key development for EEG reproducibility is the recent development of EEG-BIDS (Pernet et al., 2019) which not only structures how data are organized and shared but also populates many of the metadata necessary for data analysis. The newly developed BIDS tools^7^ used here allow to import such data and create automatically an EEGLAB STUDY with the different experimental conditions. Note that from raw data imported into EEGLAB, those tools allow just as easy to export in the BIDS compliant format. While raw EEG data files often define channel labels, EEG-BIDS defines channel properties and associated labels (channels.tsv) corresponding to the electrodes for which locations are defined (electrodes.tsv) given a reference coordinate system (coordsystem.json). BIDS-Matlab-tools will always check for consistency between the data and BIDS meta-data and users have the choice on which information to use (in Figure 2, *pop_import_bids* parameter “bidschanlocs”). Similarly, raw EEG data files typically contain behavioral and experimental events; these are also defined in BIDS with separate text files (events.tsv). Sometimes the BIDS event files contain different information than the raw EEG data file and users have options to choose which one of the two types of event information to import (in Figure 2, pop_import_bids parameter “bidsevent”). Here, when preparing the BIDS dataset with EEG data only, events.tsv file was prepared as to include the nine experimental conditions but also the repetition order, distance, and time between repetitions which allowed to create the proposed design automatically.

Data preprocessing is performed here by first cleaning the raw data using the *clean_rawdata* EEGLAB plugin (v2.2), then by running the Infomax Independent Component Analysis algorithm (*runica* function of EEGLAB 2020.0) and performing automated ICA component labeling (*ICLabel v1.2.6*). EEGLAB includes a variety of algorithms and other approaches can be implemented to preprocess data automatically. One key development for reproducible EEG artifact reduction is the use of the ICLabel EEGLAB plugin which labels independent brain and non-brain components automatically thus allowing to remove artifactual components in a consistent manner (Pion-Tonachini et al., 2019). After preprocessing, various designs can be created from a STUDY (depending upon the type and quantity of events) and LIMO tools are called to run first level analyses automatically from which group-level analyses can be performed.

The current analysis focused on ERPs for sensor space, but EEGLAB-LIMO tools can be used similarly for spectral and time-frequency analyses. Analyses may also be applied to source resolved EEG data. LIMO tools already allow using ICA components as input, since ICA components have been shown to represent activity within localized patches of cortex (Delorme et al., 2012) and future versions will also allow automatic source space analyses. Source resolved activity calculated using eLoreta, and summarized using principal component analysis within regions of interest (ROI) is also possible with the ROIconnect plugin^8^ which allows exporting activity in ROI defined in standard atlases to EEGLAB native EEG linear decomposition matrix activity (usually used for ICA activity), therefore, enabling their use in LIMO EEG.

## Data Availability Statement

Publicly available datasets were analyzed in this study. This data can be found here: https://openneuro.org/datasets/ds002718.

## Ethics Statement

The studies involving human participants were reviewed and approved by the Cambridge University Psychological Ethics Committee. The patients/participants provided their written informed consent to participate in this study.

## Author Contributions

RM-C, DT, and AD prepared the data. CP and AD wrote the code. SM overviewed the data preparation and pipeline. All authors contributed to the manuscript.

## Conflict of Interest

The authors declare that the research was conducted in the absence of any commercial or financial relationships that could be construed as a potential conflict of interest.

## Footnotes

1 https://github.com/sccn/bids-matlab-tools

2 https://www.easycap.de/

3 https://uk.mathworks.com/products/matlab.html

4 https://sccn.ucsd.edu/eeglab

5 https://github.com/sccn/bids-matlab-tools

6 https://github.com/LIMO-EEG-Toolbox/limo_tools

7 https://github.com/sccn/bids-matlab-tools

8 https://github.com/arnodelorme/roiconnect
